# OCTN2-Mediated Acetyl-l-Carnitine Transport in Human Pulmonary Epithelial Cells In Vitro

**DOI:** 10.3390/pharmaceutics11080396

**Published:** 2019-08-07

**Authors:** Johanna J. Salomon, Julia C. Gausterer, Mohammed Ali Selo, Ken-ichi Hosoya, Hanno Huwer, Nicole Schneider-Daum, Claus-Michael Lehr, Carsten Ehrhardt

**Affiliations:** 1 Department of Translational Pulmonology, Translational Lung Research Center (TLRC), Member of the German Center for Lung Research (DZL), University of Heidelberg, 69120 Heidelberg, Germany; 2 School of Pharmacy and Pharmaceutical Sciences and Trinity Biomedical Sciences Institute, Trinity College Dublin, Dublin 2, Ireland; 3 Clinical Pharmacy Department, Faculty of Pharmacy, University of Kufa, Al-Najaf 31001, Iraq; 4 Department of Pharmaceutics, Graduate School of Medicine and Pharmaceutical Sciences, University of Toyama, Toyama 930-0194, Japan; 5 Department of Cardiothoracic Surgery, Völklingen Heart Centre, 66333 Völklingen, Germany; 6 Drug Delivery, Helmholtz Institute for Pharmaceutical Research Saarland (HIPS), Helmholtz Centre for Infection Research, 66123 Saarbrücken, Germany

**Keywords:** organic cation transporter, OCTN2, lung epithelium, acetyl-l-carnitine, epithelial transport, asthma, in vitro models

## Abstract

The carnitine transporter OCTN2 is associated with asthma and other inflammatory diseases. The aims of this work were (i) to determine carnitine uptake into freshly isolated human alveolar type I (ATI)-like epithelial cells in primary culture, (ii) to compare the kinetics of carnitine uptake between respiratory epithelial in vitro cell models, and (iii) to establish whether any cell line was a suitable model for studies of carnitine transport at the air-blood barrier. Levels of time-dependent [^3^H]-acetyl-l-carnitine uptake were similar in ATI-like, NCl-H441, and Calu-3 epithelial cells, whereas uptake into A549 cells was ~5 times higher. Uptake inhibition was more pronounced by OCTN2 modulators, such as l-Carnitine and verapamil, in ATI-like primary epithelial cells compared to NCl-H441 and Calu-3 epithelial cells. Our findings suggest that OCTN2 is involved in the cellular uptake of acetyl-l-carnitine at the alveolar epithelium and that none of the tested cell lines are optimal surrogates for primary cells.

## 1. Introduction

Organic cation transporters (i.e., OCT1, OCT2, and OCT3, encoded by *SLC22A1–3*) and novel organic cation transporters (i.e., OCTN1 and OCTN2, encoded by *SLC22A4–5*) play important roles in the (patho-)physiological membrane transport of endogenous and exogenous substrates, including drugs [1,2]. Evidence stemming from various pre-clinical in vitro, ex vivo, and in vivo studies regarding the involvement of organic cation transporters in pulmonary drug disposition has been published in recent years [3,4,5,6]. In addition, OCT/Ns have been identified as potential targets in lung disease. Genetic studies revealed that variants of OCTN2 are associated with asthma [7,8]; however, expression of OCTN1 and OCNT2 was not altered in lung tissues from patients with chronic obstructive pulmonary disease (COPD) [9]. 

Due to the relevance of respiratory OCTN2 in health and disease, data on its expression, localisation [10,11,12,13,14,15], and activity [6,16,17] in the lung are emerging. Generally, expression of OCTN2 was found throughout respiratory epithelium, with a higher abundance in the upper lung [13,14,18,19]. Localisation of OCTN2 was detected at the luminal membranes of airway epithelial cells and in alveolar macrophages [9,20,21]. In vivo data showed that tracheal accumulation of the anticholinergic bronchodilator ipratropium was inhibited by both carnitine and 1-methyl-4-phenylpyridinium (MPP^+^), hence OCTN2 (and probably OCT2) involvement in the process was suggested [6]. In addition, absorption of ipratropium and tiotropium was found to be augmented in OCTN2-transfected HEK-293 cells [16]. Further, challenge with lipopolysaccharide (LPS) and house dust mites significantly upregulated OCTN2 expression in Calu-3 cells in vitro, suggesting that allergic airway inflammation impacts the inhaled drug disposition via OCTN2 [22]. Al-Jayyoussi et al. showed that OCT/Ns were involved in the cellular accumulation of the substrate ipratropium and l-carnitine in human respiratory epithelial cell models; however, no carnitine-sensitive pulmonary absorption was observed using an intact, isolated, perfused rat-lung model [23]. 

Alveolar epithelial transport is generally tightly regulated [24] and our lab previously reported the expression of OCTN2 in a number of human respiratory epithelial cell lines and in primary alveolar epithelial cells [14,15]. The question of whether OCTN2 is functionally active at the alveolar epithelium remains unanswered. It was the aim of this study to investigate OCTN2 activity in primary alveolar epithelial cells using the specific OCTN2 substrate acetyl-l-carnitine ([^3^H]-AC) [25,26]. The obtained data was compared to cell lines of human respiratory epithelial origin (i.e., the bronchial mucosal gland cell line Calu-3, the distal lung epithelial cell line NCl-H441, and the alveolar epithelial cell line A549) [3,27] to establish if any cell line was a useful model to study alveolar epithelial OCTN2 function in vitro.

## 2. Materials and Methods

### 2.1. Materials

[N-methyl-^3^H] acetyl-l-carnitine hydrochloride ([^3^H]-AC; 85 Ci/mmol) was purchased from American Radiolabeled Chemicals Limited (Herts, UK). Chemicals and all cell culture media and supplements were obtained from Sigma-Aldrich (Dublin, Ireland, or St. Louis, MO, USA), with the exception of ergothioneine, which was bought from Santa Cruz Biotechnology (Heidelberg, Germany). All cell culture plastics were obtained from Greiner BioOne (Frickenhausen, Germany). 

### 2.2. Human Alveolar Epithelial Cell Isolation and Culture

The use of human tissue specimens was approved by Saarland State Medical Board (Saarbrücken, Germany). Human alveolar type II epithelial cells were isolated from non-tumor lung tissue obtained from patients undergoing lung surgery as previously published [28,29]. Purified type II cells were seeded at a density of 400,000 cells/cm^2^ on collagen/fibronectin-coated plastics using complete small airways growth medium (SAGM; Lonza, Verviers, Belgium) supplemented with 1% foetal bovine serum (FBS), 100 U/mL penicillin, and 100 µg/mL streptomycin. Primary cell monolayers were used after transdifferentiation into an alveolar type I-like (ATI-like) phenotype following at least one week of culture.

### 2.3. Cell Line Culture

A549 epithelial cells (ATCC CCL-185) were obtained from the European Collection of Animal Cell Cultures (Salisbury, UK). Calu-3 (ATCC HTB-55) and NCI-H441 (ATCC HTB-174) cells were purchased from LGC Promochem (Teddington, UK). Cell lines were cultured as previously published [15,30]. All cell lines were maintained at 37 °C in 5% CO_2_ atmosphere and the culture media were exchanged every other day. 

### 2.4. Uptake Studies 

Uptake experiments using A549 epithelial cells were carried out in extracellular fluid buffer (ECF; 122 mM NaCl, 3 mM KCl, 0.4 mM KHPO_4_, 25 mM NaHCO_3_, 1.4 mM CaCl_2_, 1.2 mM MgSO_4_, 10 mM 4-(2-hydroxyethyl)-1-piperazineethanesulfonic acid (HEPES), and 10 mM d-glucose, pH 7.4). All other experiments were performed in freshly prepared, bicarbonated Krebs–Ringer buffer (KRB; 116.4 mM NaCl, 5.4 mM KCl, 0.78 mM NaH_2_PO_4_, 25 mM NaHCO_3_, 1.8 mM CaCl_2_, 0.81 mM MgSO_4_, 15 mM HEPES, and 5.55 mM d-glucose, pH 7.4), unless otherwise stated. Both buffer solutions were found not to be significantly different with regard to organic cation uptake as previously reported [30] and organic carnitine uptake (data not shown). To initiate uptake, 200 μL of buffer solution containing [^3^H]-AC (5.5 nM) was added to each well. For time-course analyses, uptake was studied over 30 min at 37 °C. To determine concentration dependency and (self)-inhibitory effects on acetyl-l-carnitine uptake, cell monolayers were incubated with acetyl-l-carnitine in the presence of various concentrations (i.e., 0–1 mM) of unlabeled compound for 20 min. In this case, [^3^H]-AC uptake was carried out at 4 °C and 37 °C and values obtained at 4 °C were subtracted from values measured at 37 °C in order to account for adsorption and diffusion processes. In all studies, a concentration of 5.5 nM [^3^H]-AC was used. Uptake of acetyl-l-carnitine was also performed in the presence of several modulators of organic carnitine and organic cation transporter function (i.e., 4-(4-(dimethylamino)styryl)-N-methyl-pyridinium iodide (ASP^+^), corticosterone, d-carnitine, ergothioneine, MPP^+^, quinidine, and verapamil). At the relevant time points, uptake was stopped by washing cell monolayers three times with ice-cold buffer and 400 µL of 1 N NaOH was added to permeabilise the cells for at least 12 h, before 400 µL of 1 N HCl was used for neutralisation of the cell lysate. Five-hundred microliters of lysate was used to measure the cell-associated radioactivity in a liquid scintillation counter (Tri Carb TR2100 Packard Scintillation Counter, Dublin, Ireland). In parallel, the total cell protein content was quantified using a DC^TM^ Protein Assay kit (Bio-Rad, Hemel Hempstead, UK) according to the manufacturer’s instructions.

### 2.5. Transport Studies

Cells were grown on Transwell Clear inserts for at least 8 (NCI-H441) or 14 (Calu-3) days. To initiate transport studies, both sides of the cultured cell layers were washed twice with pre-warmed KRB solution, followed by a 60 min equilibration in KRB at 37 °C. The initial donor concentration was determined by taking a 10 µL sample directly after adding the donor solution. The cell layers were kept at 37 °C during experiments and 200 µL samples were collected from the receiver compartment at the designated time points from the respective compartments (the radioactivity of the samples was determined with a Tri Carb TR2100 Packard Scintillation Counter, Ireland). To keep sink conditions, an equal amount of fresh pre-warmed KRB solution was returned to the receiver compartments. At the end of transport studies, another 10 µL sample was collected from the donor compartment. Each experiment was conducted at least in triplicates. Transepithelial electrical resistance (TEER) values were recorded before and after the flux studies, in order to assess the cell layer integrity.

The following equation was used to calculate the apparent permeability coefficient (P_app_):

P_app_ = (ΔQ/Δt)/(A × C_0_), (1)

where ΔQ is the change in amount of the compound over a designated period of time (Δt), A is the nominal surface area of the cell layers (1.13 cm^2^), and C_0_ is the initial concentration of the drug in the donor fluid used in this study.

### 2.6. Data Analysis

The uptake of [^3^H]-AC by human respiratory epithelial cells was expressed as the cell-to-medium (cell/medium) ratio, which was calculated by the following equation [31]:

Cell/medium ratio = [^3^H] dpm per mg cell protein/[^3^H] dpm per μL buffer. (2)

To estimate the kinetic uptake parameters of [^3^H]-AC, the initial uptake rates were fitted to Equation (3) by means of non-linear least-squares regression analysis using WinNonlin (Pharsight, Sunnyvale, CA, USA).

v = V_max_ × s/K_m_ + s, (3)

where v is the initial uptake rate of the substrate (nmol/(min/mg protein)), s is the substrate concentration in the medium (μM), K_m_ is the Michaelis–Menten constant (μM), and V_max_ is the maximum uptake rate (nmol/min/mg protein).

The time-dependent uptake was fitted using SigmaPlot 12.5 (Systat Software, Erkrath, Germany). One binding site was assumed and fitting was corrected for non-specific binding. Data were analyzed with SigmaPlot 12.5 (Systat Software, Erkrath, Germany) and shown as mean ± standard deviation (SD). Specifically for inhibition experiments, results were expressed as mean ± standard error of the mean (SEM). Statistical analysis was performed using unpaired and paired two-tailed Student’s *t*-test and one-way analysis of variance (ANOVA), as appropriate, and *p*-values < 0.05 were accepted to indicate statistical significance. All data were obtained from at least 3 independent experiments.

## 3. Results

### 3.1. Time Course of Acetyl-l-Carnitine Uptake by Human Respiratory Epithelial Cells

**Figure 1:**
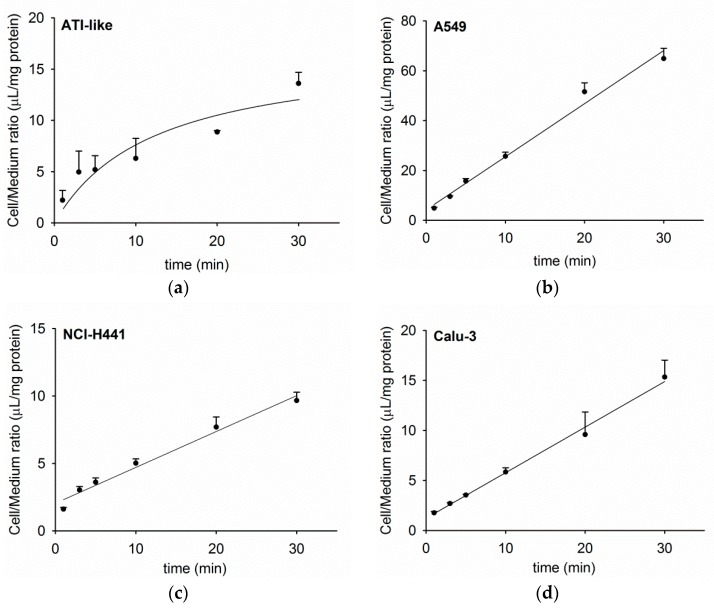
Time course of [^3^H]-AC uptake into human alveolar (**a**) alveolar type I (ATI)-like, (**b**) A549, (**c**) bronchiolar (NCl-H441), and (**d**) bronchial (Calu-3) epithelial cell types. Cell uptake of [^3^H]-AC was performed at 37 °C for 30 min and data were fitted by means of non-linear regression analysis. Each point represents mean ± SD (*n* = 3).

The time course of acetyl-l-carnitine uptake into human lung epithelial cell monolayers is shown in Figure 1a–d. Uptake of [^3^H]-AC into primary ATI-like cells increased to 13.6 ± 1.1 µL/mg protein (Figure 1a). A similar maximal cell/medium ratio of [^3^H]-AC uptake was observed in NCl-H441 (9.7 ± 0.6 µL/mg protein) and Calu-3 epithelial cells (15.3 ± 1.7 µL/mg protein, Figure 1c,d). In the case of A549 cells, the linear uptake of [^3^H]-AC reached 64.9 µL/mg protein after 30 min (Figure 1b).

### 3.2. Concentration Dependence of Acetyl-l-Carnitine Uptake

**Figure 2:**
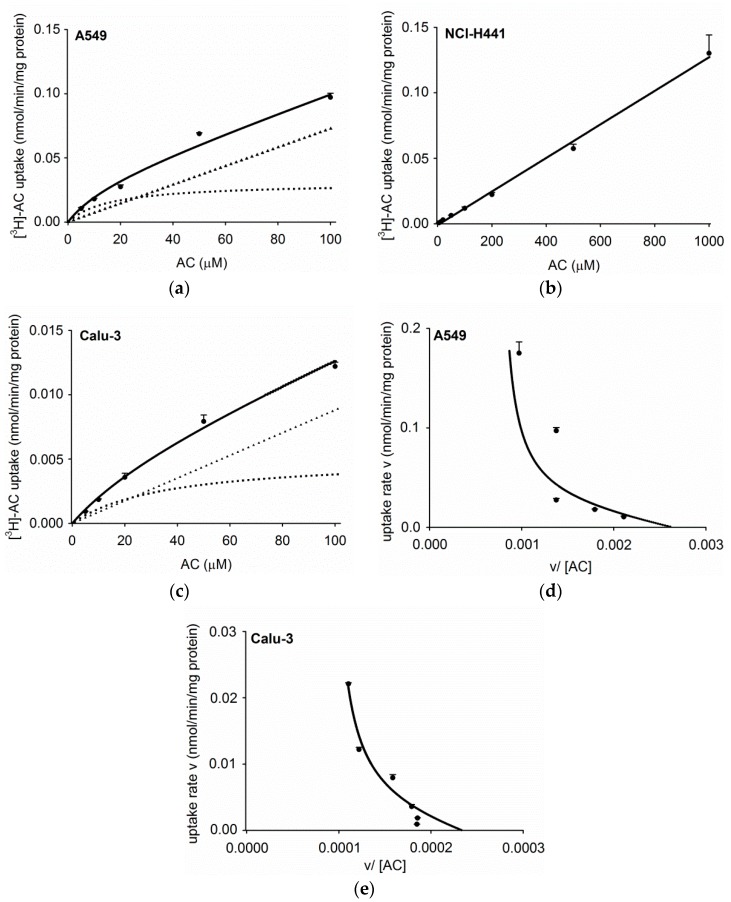
Concentration dependence of [^3^H]-AC uptake by (**a**) A549, (**b**) NCl-H441, and (**c**) Calu-3 epithelial cells for 20 min. Furthermore, [^3^H]-AC uptake was analyzed by means of Eadie–Hofstee equation in the case of (**d**) A549 and (**e**) Calu-3 cells, respectively: v, uptake rate (nmol/min/mg protein); [AC], acetyl-l-carnitine concentration in µM; v/[AC] (µL/min/mg protein). Total (solid line), saturable part (broken line), and non-saturable part (triangle) of [^3^H]-AC uptake is shown up to 100 µM, calculated by using the kinetic parameters *K*_m_, V_max_, and *K*_d_ for (**d**) A549 and (**e**) Calu-3 epithelial cells. These results were obtained by subtraction of [^3^H]-AC uptake at 4°C as the non-specific component from the total uptake. Data represent mean ± SD (*n* = 3).

The concentration dependence of acetyl-l-carnitine uptake was examined in the three human respiratory epithelial cell lines in more detail. In A549 monolayers, a saturable uptake of [^3^H]-AC was observed (Figure 2a) and revealed a *K*_m_ value of 16.1 ± 10.9 µM, a V_max_ of 0.03 ± 0.01 nmol/min/mg protein, and a non-saturable transport clearance value (*K*_d_) of 0.7 µL/min/mg protein (Figure 2d). In the case of NCl-H441 cell monolayers, no saturation of [^3^H]-AC uptake was obtained (Figure 2b). In Calu-3 cell monolayers, [^3^H]-AC uptake was found to be saturable (Figure 2c) with a *K*_m_ = 35.4 ± 26.7 µM, V_max_ = 0.005 ± 0.003 nmol/min/mg protein, and *K*_d_ of 0.1 µL/min/mg protein (Figure 2e).

### 3.3. Influence of Pharmacological Modulators on Acetyl-l-Carnitine Uptake

**Figure 3:**
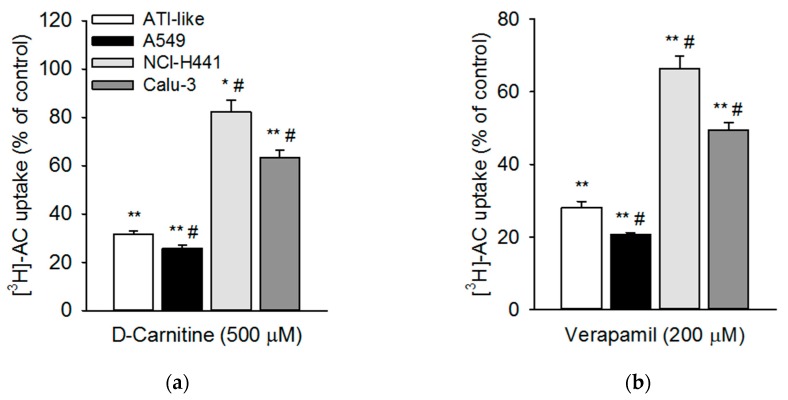
Effect of OCTN2-modifying pharmacophores on the uptake of [^3^H]-AC into human respiratory epithelial cell monolayers. Uptake of [^3^H]-AC into ATI-like (white columns), A549 (black columns), NCl-H441 (light grey columns), and Calu-3 (dark grey columns) epithelial cells was measured after 20 min at 37 °C and at pH 7.4 in the presence of (**a**) d-carnitine (500 µM) and (**b**) verapamil (200 µM). The results were obtained by calculating the difference between uptake at 37 °C and 4 °C. Data represent mean ± SEM (*n* = 3–6). ** *p* < 0.01; * *p* < 0.05 indicates a significant difference from the uptake at pH 7.4 (control). # *p* < 0.05 indicates a significant difference from data obtained in ATI-like monolayers.

The effects of two known pharmacological OCTN2 modulators on [^3^H]-AC uptake by human respiratory epithelial cells and ATI-like epithelial cell monolayers were studied, i.e., d-carnitine and verapamil. Concentrations of modulators were chosen according to their known inhibitory potential [32]. The uptake of [^3^H]-AC into ATI-like epithelial cells was inhibited to approximately 30% of the control (*p* < 0.01) in the presence of d-carnitine and verapamil (Figure 3). Similar inhibition was observed when measuring [^3^H]-AC uptake into A549 cells. Here, d-carnitine and verapamil comparably reduced uptake to 25.8% ± 1.3% and 20.7% ± 0.5%, respectively. In NCl-H441 and Calu-3 cells, the effects of the OCTN2 inhibitors on [^3^H]-AC uptake were much less pronounced (i.e., 20%50% inhibition).

**Figure 4:**
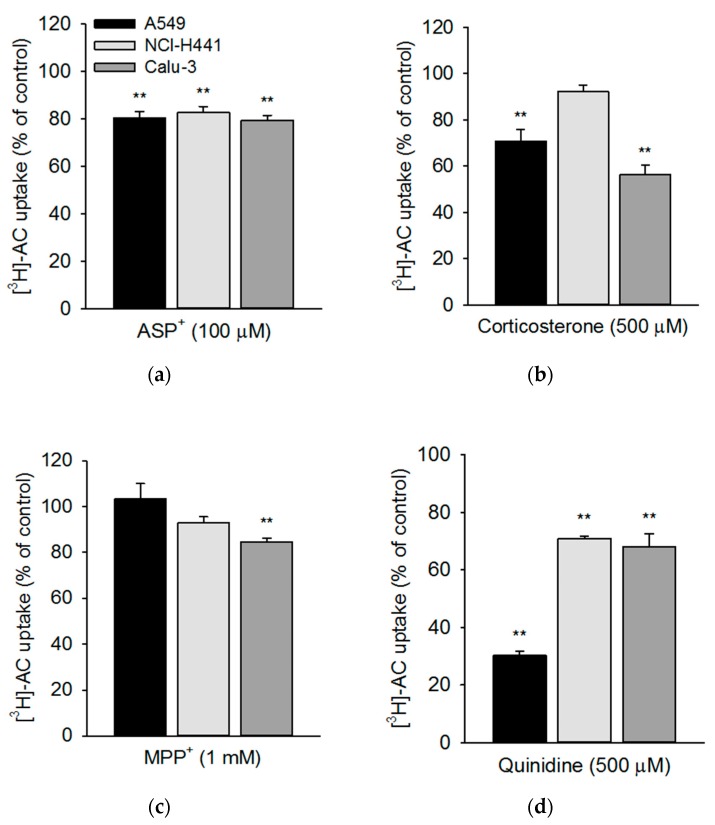
[^3^H]-AC uptake was studied in the presence and absence of OCT/N modulators in human respiratory epithelial cells. [^3^H]-AC uptake was studied in A549 (black columns), NCl-H441 (light grey columns), and Calu-3 (dark grey columns) cells after 20 min in the presence of (**a**) ASP^+^ (100 µM), (**b**) corticosterone (500 µM), (**c**) MPP^+^ (1 mM), and (**d**) quinidine (500 µM) at 37 °C and at pH 7.4. Results were obtained by calculating the difference between uptake at 37 °C and 4 °C. Data represent mean ± SEM (*n* = 6–9). ** *p* < 0.01 indicates a significant difference from data obtained in the absence of substances (control).

In a subset of experiments, a larger panel of modulators of OCT/N function was tested in the three continuously grown epithelial cell lines (Figure 4a–d). We selected the pan-OCT substrate MPP^+^, the OCT2/OCTN substrate ASP^+^, the OCT1-3/OCTN2 inhibitor corticosterone, and OCT1/OCTN inhibitor quinidine to pharmacologically characterise OCTN2-mediated acetyl-l-carnitine uptake [14,25]. Co-incubation with the ASP^+^ resulted in a weak, yet significant, inhibition of [^3^H]-AC uptake in all three cell lines at a very similar level (Figure 4a). Corticosterone affected the [^3^H]-AC uptake in A549 and Calu-3 cell layers, but no effect was observed in NCl-H441 cells (Figure 4b). MPP^+^ had almost no effect on the uptake of [^3^H]-AC in the three respiratory epithelial cell lines, with inhibitory effects only significant in Calu-3 cells (Figure 4c). Co-incubation with quinidine resulted in a strong inhibition of [^3^H]-AC uptake (up to 70%) in A549 cells (Figure 4d), with lesser effects in NCI-H441 and Calu-3 cells.

### 3.4. Transport of Acetyl-l-Carnitine Uptake across NCl-H441 and Calu-3 Cells

Bidirectional transport studies were performed using Transwell Clear-grown NCl-H441 and Calu-3 cell monolayers. Transport of acetyl-l-carnitine across NCl-H441 cell monolayer showed no net directionality (*P*_app_ (a to b) = 3.6 ± 0.2 × 10^−6^ cm/s and *P*_app_ (b to a) = 4.3 ± 0.3 × 10^−6^ cm/s). Similarly, transport of [^3^H]-AC across Calu-3 monolayers revealed no net direction (*P*_app_ (a to b) 1.3 ± 0.1 × 10^−6^ cm/s and *P*_app_ (b to a) 1.3 ± 0.1 × 10^−6^ cm/s), however, transport significantly decreased 3-fold when compared to NCl-H441 cell monolayers (*p* < 0.01).

## 4. Discussion

Single nucleotide polymorphisms (SNPs) in the *SLC22A5* gene, which encodes OCTN2, have consistently been associated with asthma and other inflammatory diseases [33] and in vitro results implicate an upregulation of OCTN2 expression by aeroallergens [22]. At the same time, the role of uptake transporters in pulmonary drug disposition is far from being understood [34]. Interestingly, pulmonary administration of an OCTN2-transported carnitine ester prodrug of prednisolone showed reductions in signs of airway inflammation in an asthma guinea pig model [35]. Therefore, in this work, we sought to determine the activity of OCTN2 at the alveolar epithelial barrier in vitro. In search for a suitable in vitro model for studying OCTN2 at the human alveolar epithelial barrier, we further investigated similarities and differences in OCTN2 activity between ATI-like primary cells and respiratory epithelial cell lines (i.e., A549, NCl-H441, and Calu-3).

Our results confirmed a time-dependent uptake of acetyl-l-carnitine into primary alveolar epithelial cells, which exhibited a similar uptake compared to bronchial (Calu-3) and bronchiolar (NCl-H441) epithelial cell lines. Uptake of acetyl-l-carnitine into the alveolar epithelial cell line, A549, was considerably higher. Kinetic analysis of acetyl-l-carnitine uptake showed *K_m_* values of 17.7 µM and 35.4 µM in A549 and Calu-3 cells, respectively, which was in good agreement with results from the literature [17]. d-carnitine (OCTN2 inhibitor) and verapamil (OCT/N inhibitor) strongly inhibited acetyl-l-carnitine uptake in primary human ATI-like alveolar epithelial cells and the A549 cell line, whereas lower levels of inhibition were observed in NCl-H441 and Calu-3 cells. ASP^+^, corticosterone, and MPP^+^, which are all known as ubiquitous OCT substrates, showed marginal inhibitory effects (less than 40%), confirming the specificity of OCTN2-mediated cellular acetyl-l-carnitine uptake into respiratory epithelial cells [23]. Furthermore, the rank order of inhibitory effect of the OCT substrates (corticosterone > ASP^+^ > MPP^+^) was in good agreement with lower IC_50_ (concentration needed to inhibit 50% of maximum effect) values reported for corticosterone than MPP^+^ in OCTN2-expression systems [25]. Quinidine was reported as a mixed OCT1/OCTN substrate [25] and thus blocked more potent acetyl-l-carnitine uptake in the respiratory epithelial cell models (30%70% inhibition). These findings confirmed previous results from studies in OCTN2-transfected HEK-293 cells [36,37].

Bidirectional acetyl-l-carnitine transport studies across Calu-3 and NCl-H441 epithelial cell monolayers revealed no net absorption of the substrate in both models. It was shown that carnitine actively accumulated by organic cation/carnitine transporters in the trachea in vivo [6], but in the absence of a basolateral efflux transporter for carnitine, tissue accumulation did not result in increased systemic absorption. This was in agreement with data from Al-Jayyoussi et al., who reported that l-carnitine transport in an intact, isolated, perfused rat-lung model was not impacted by pre-administration of l-carnitine [23]. Physiologically, the cellular uptake of carnitine by OCTN2 is essential for carnitine homoeostasis, which is achieved by endogenous biosynthesis (mainly in the liver and kidney), intake from the diet, and renal reabsorption [38]. Carnitine is essential for β-oxidation of long-chain fatty acids in mitochondria [38,39]. In murine alveolar epithelium, it was proposed that when fatty acid oxidation is impaired, pulmonary surfactant levels and lung function is decreased [40]. Acylcarnitines, which are catabolised from carnitine, directly inhibit the activity of alveolar surfactant [40]. Importantly, the level of acylcarnitine is measured when screening for fatty acid oxidation disorders in newborns [41].

## 5. Conclusions

Acetyl-l-carnitine uptake, but not translocation, at the human alveolar epithelial barrier is mediated by OCTN2. The A549 cell line showed a similar profile to ATI-like primary cells with regard to pharmacological inhibition; however, [^3^H]-AC uptake kinetics were distinctively different. NCl-H441 and Calu-3 cell lines, on the other hand, showed closer kinetic similarity, but vastly different inhibitor profiles. Thus, none of these cell lines can be recommended as a surrogate for studying OCTN2 function at the human alveolar epithelial barrier in vitro. Moreover, the number of inhibitors to study the specific uptake of acetyl-l-carnitine at the airway epithelium is rather limited. Novel inhibitors and/or activators are needed to further elaborate the function of SLC22 transporter proteins to evaluate their potential as targets in therapeutic approaches [42].
